# Immunodetection of some pectic, arabinogalactan proteins and hemicellulose epitopes in the micropylar transmitting tissue of apomictic dandelions (*Taraxacum*, Asteraceae, Lactuceae)

**DOI:** 10.1007/s00709-016-0980-0

**Published:** 2016-05-06

**Authors:** Robert Gawecki, Katarzyna Sala, Ewa U. Kurczyńska, Piotr Świątek, Bartosz J. Płachno

**Affiliations:** 1 Department of Cell Biology, Faculty of Biology and Environmental Protection, University of Silesia, 28 Jagiellońska St., 40-032 Katowice, Poland; 2 Department of Animal Histology and Embryology, University of Silesia, 9 Bankowa St., 40-007 Katowice, Poland; 3 Department of Plant Cytology and Embryology, Jagiellonian University in Kraków, 9 Gronostajowa St., 30-387 Kraków, Poland

**Keywords:** Ovule, Transmitting tissue, Gynoecium, Dandelions, Asteraceae, Apomixis, Arabinogalactan proteins, Pectins, Hemicellulose, *Taraxacum*

## Abstract

In apomictic *Taraxacum* species, the development of both the embryo and the endosperm does not require double fertilisation. However, a structural reduction of ovular transmitting tissue was not observed in apomictic dandelions. The aim of this study was to analyse the chemical composition of the cell walls to describe the presence of arabinogalactan proteins (AGPs), hemicellulose and some pectic epitopes in the micropylar transmitting tissue of apomictic *Taraxacum*. The results point to (1) the similar distribution of AGPs in different developmental stages, (2) the absence of highly methyl-esterified homogalacturonan (HG) in transmitting tissue of ovule containing a mature embryo sac and the appearance of this pectin domain in the young seed containing the embryo and endosperm, (3) the similar pattern of low methyl-esterified pectin occurrence in both an ovule and a young seed with an embryo and endosperm in apomictic *Taraxacum* and (4) the presence of hemicelluloses recognised by LM25 and LM21 antibodies in the reproductive structure of *Taraxacum*.

**Electronic supplementary material:**

The online version of this article (doi:10.1007/s00709-016-0980-0) contains supplementary material, which is available to authorized users.

## Introduction

The stylar and ovule transmitting tissues form the environment and guidance for the growth of a pollen tube (male gametophyte) into a female gametophyte. The transmitting tissue is composed of a stigma, style and the adaxial carpel epidermis within the ovary locule and, frequently, a funiculus (forming obturator) (e.g. Vasil and Johri 1964; Tilton and Horner 1980; Lyew et al. 2007). The stylar transmitting tissue is anatomically and histochemically similar to the dry stigma (Sage et al. 2009). The most prominent character of this tissue is the extracellular matrix (ECM), which consists of intercellular spaces filled with a secretion as well as the walls of the transmitting tissue cells (Lord 2003). From an evolutionary perspective, the presence of a well-developed transmitting tissue in the basal angiosperms such as *Amborella trichopoda*, *Illicium floridanum*, *Trimenia moorei* and *Acorus americanus* suggests its ancient role in the evolution of flowering plants (Sage et al. 2009). The ECM contains compounds (including calcium, lipids, arabinogalactan proteins (AGPs), a lipid-like transfer proteins and methyl-esterified homogalacturonan), which facilitate, and also guide, the adhesion and growth of the pollen tubes (e.g. Wu et al. 2000; Lord and Russell 2002; Khosravi et al. 2003; Lenartowska et al. 2001; Coimbra and Duarte 2003; Sage et al. 2009). AGPs are not only involved in sexual plant reproduction but may also be useful markers for gametophytic cell differentiation (e.g. Coimbra and Salema 1997; Coimbra and Duarte 2003; Coimbra et al. 2007, 2008; Wiśniewska and Majewska-Sawka 2006; Rafińska and Bednarska 2011; Chudzik et al. 2014) and ovule receptivity (Coimbra and Duarte 2003; Chudzik 2002; Śnieżko and Chudzik 2003; Chudzik et al. 2005b). In fertile and mature angiosperm ovules, epitopes that were recognised by the JIM8 or JIM13 antibodies were detected on the micropylar pole in the tissues lying on the pathway of pollen tube growth (Coimbra and Salema 1997; Śnieżko and Chudzik 2003; Chudzik et al. 2005b; Coimbra et al. 2007); thus, the AGPs recorded in this localisation may act as lubricants and/or nutrients for the pollen tube (Suárez et al. 2013). Similar to AGPs, an accumulation of homogalacturonan in both esterified and unesterified forms was found in the tissues lying on the pathway of the pollen tube growth only in fertile and mature ovules (Chudzik 2002; Śnieżko and Chudzik 2003; Niedojadło et al. 2015). The question is whether the accumulation of arabinogalactan proteins and homogalacturonan occurs in the micropyle of obligatory apomicts, in which the development of both the embryo and the endosperm does not require double fertilisation. To date, there is only one species that has been examined from this group—*Chondrilla juncea* L. (Kościńska-Pająk et al. 2005; Kościńska-Pająk 2006; Chudzik et al. 2005a). According to these authors, both esterified and deesterified pectins were abundant in the micropylar canal matrix but were not present in the micropylar part of the embryo sac. Moreover, there was a lack of AGP epitopes that were recognised by the JIM13 antibody inside the micropylar canal and mature embryo sac in *C. juncea*. Thus, the occurrence of AGPs and homogalacturonan has mostly been determined in the ovules in amphimictic species and data about apomicts are fragmentary and unsatisfactory. Recently, the anatomy and ultrastructure of the transmitting tissue was analysed in apomictic *Taraxacum* species, and no major differences were detected between the micropyle structure of the amphimictic and apomictic *Taraxacum* (Płachno et al. 2015a, b).

We would like to highlight that over 24,000 species are recognised in the Asteraceae family (Funk et al. 2009), but so far, the immunohistochemistry of the cell walls in the ovule tissue/embryo sac has been described only for two species (*C. juncea* and *Bellis perennis*) from this family. Therefore, the aim of our study was to check whether and which AGPs, pectic and hemicellulose epitopes occur in the micropylar transmitting tissue of apomictic *Taraxacum*. None of the studies on Asteraceae family gives as a full picture of immunochemistry of cell walls in micropylar transmitting tissue and synergids as we present here on the example of *Taraxacum*. Such knowledge about the chemical composition of cell walls or its changes might be used (in the future) to explain the mechanisms underlying different ways of reproduction (apomictic and sexual).

## Material and methods

**Fig. 1:**
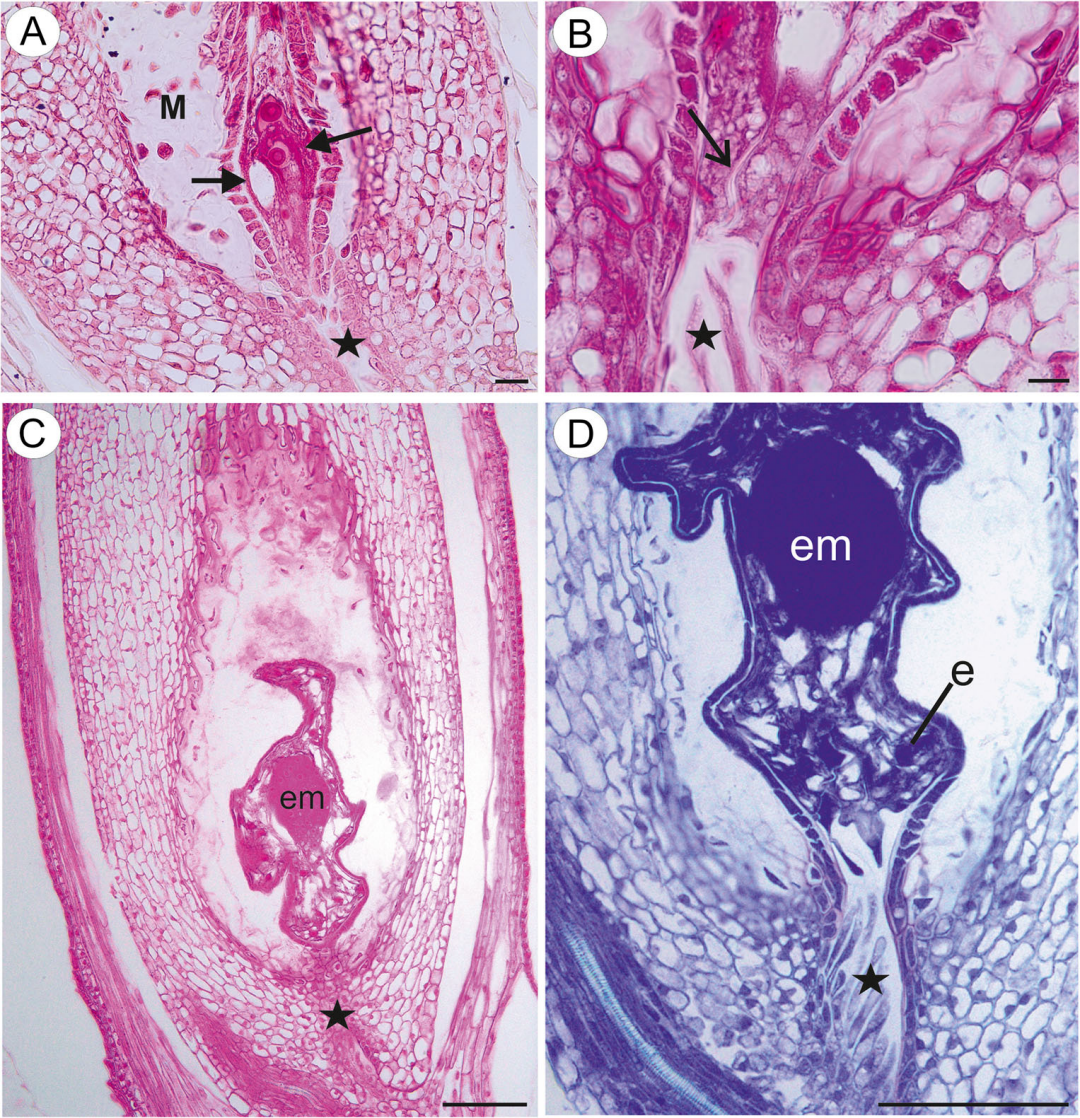
Section through the ovule and young seed of *Taraxacum* showing the developmental stages. **a**, **b** Section through the ovule from a flower harvested before anthesis, showing a mature embryo sac with the egg apparatus (*black arrows*), synergid filiform apparatus (*open arrow*), micropylar transmitting tissue cells (*black star*) and mucilage cells (*M*), *bar* = 20 μm. **c**, **d** Section through a young seed from a flower harvested during anthesis, showing the embryo (*em*), endosperm (*e*) and micropylar transmitting tissue cells (*black star*), *bar* = 100 μm

The material was represented by apomictic taxa from the *Taraxacum* sect. *Taraxacum* (*Taraxacum officinale* agg., collected in Katowice, Silesia, Poland, and also from the collection of the Department of Plant Cytology and Embryology, Jagiellonian University). Studies were carried out on flowers before and during anthesis. The flowers of the apomictic species harvested before anthesis contained a mature embryo sac (Fig. 1a, b), whereas the flowers harvested during anthesis already contained an embryo and endosperm (Fig. 1c, d) as it was previously observed (Płachno et al. 2014; Płachno et al. 2015a, b).

Individual ovaries were collected with a razor blade under an Olympus SZH10 stereomicroscope and fixed in a mixture of 3 % (*w*/*v*) paraformaldehyde (PFA), 1.25 % (*v*/*v*) glutaraldehyde (GA), 1 % (*w*/*v*) sucrose and 0.1 % (*w*/*v*) Triton X-100 in phosphate-buffered saline (PBS; pH 7.4). To remove the air from the material and to facilitate fixative infiltration, samples were put under a vacuum for 5 × 15 min bursts. The samples were fixed overnight at 4 °C and then rinsed with PBS (3 × 15 min), dehydrated in an ethanol series (10, 30, 50, 70, 90 and 100 %, *v*/*v*) and embedded in Steedman’s wax according to Vitha et al. (2000). Material was cut into 7-μm-thick sections using a Zeiss Hyrax M40 microtome. Sections were collected on microscopic slides covered with Mayer’s adhesive albumin (Potocka et al. 2012; Sala et al. 2013). Before staining procedures, the sections were dewaxed and rehydrated in a successive ethanol series (three times in 100, 90 and 50 % in PBS (*v*/*v*), 10 min each).

### Histochemistry

For general histology, the sections were stained with a 0.05 % aqueous solution of Toluidine Blue O for 5 min. A 0.02 % aqueous solution of ruthenium red was used to detect pectin (10 min staining) and a 0.02 % aqueous solution of neutral red to confirm the presence of mucilage (5 min staining). All slides were mounted with a 50 % (*v*/*v*) aqueous glycerol solution, observed and photographed with a Nikon Eclipse Ni-U microscope equipped with a Nikon Digital DS-Fi1-U3 camera (Nikon, Tokyo, Japan).

### Immunocytochemistry

Sections were blocked in a buffer containing 2 % (*v*/*v*) foetal calf serum (FCS) and 2 % (*w*/*v*) bovine serum albumin (BSA) in PBS (pH 7.2, room temperature (RT), 30 min). Next, the sections were incubated with primary monoclonal antibodies (see Table 1), diluted 1:20 in a blocking buffer (RT, minimum 1.5 h), rinsed with the blocking buffer (three times, 10 min each) then incubated with the secondary antibody (Alexa Fluor 488 goat anti-rat IgG, Jackson Immuno-Research Laboratories, West Grove, USA) and diluted 1:100 in the blocking buffer (RT, minimum 1.5 h). After washing (three times, 10 min each) with the blocking buffer and PBS, the sections were stained with 0. 01 % (*w*/*v*) fluorescent brightener 28 (Sigma, St. Louis, USA) in PBS for 5 min, rinsed with PBS and distilled sterile water (four times, 5 min for each), shaken dry and mounted with a Fluoromount (Sigma, St. Louis, USA) anti-fading medium. Some sections were stained with both fluorescent brightener 28 (Sigma, St. Louis, USA) and with DAPI (4′,6-diamidino-2-phenylindole; Invitrogen Inc.; 2 μg/ml, RT, 5 min). Negative controls were performed by omitting the primary antibody step, obtaining no fluorescence signal in each control frame for all slides stained.

**Table 1:** List of the monoclonal antibodies used in the current study, the epitopes they recognised and references

Antibody	Epitope	References
Hemicelluloses		
LM21	β-(1→4)-Manno-oligosaccharides from mannan, glucomannan and galactomannan	Marcus et al. 2010
LM25	XLLG, XXLG and XXXG motifs of xyloglucan	Pedersen et al. 2012
Pectins		
LM19	HG domain in pectic polysaccharides, recognises a range of HG with the preference to bind strongly to unesterified HG	Verhertbruggen et al. 2009, www.plantprobes.net
LM20	HG domain in pectic polysaccharides, requires methyl esters for recognition of HG and does not bind to unesterified HG	Verhertbruggen et al. 2009, www.plantprobes.net
LM8	Xylogalacturonan (HG domain)	Willats et al. 2004
JIM5	HG domain of pectic polysaccharides, recognises partially methyl-esterified epitopes of HG, can also bind to unesterified HG	Knox et al. 1990, www.plantprobes.net
JIM7	HG domain of pectic polysaccharides, recognises partially methyl-esterified epitopes of HG but does not bind to unesterified HG	Knox et al. 1990, www.plantprobes.net
AGPs		
JIM8	Arabinogalactan	Pennell et al. 1991
JIM13	Arabinogalactan/arabinogalactan protein	Knox et al. 1991
JIM16	Arabinogalactan/arabinogalactan protein	Knox et al. 1991

Observations and photography were carried out with a Nikon Eclipse Ni-U microscope (or an Olympus BX60 microscope) equipped with a Nikon Digital DS-Fi1-U3 camera with corresponding software (Nikon, Tokyo, Japan) and filters for Alexa Fluor 488 (excitation filter 450-490, barrier filter BA520), fluorescent brightener 28 and DAPI (excitation filter 330-380, barrier filter BA420). Sections labelled with the JIM13 antibody were also imaged using an Olympus Fluoview FV1000 laser scanning microscope. The excitation wavelength for the Alexa Fluor 488 was 488 nm, and the emission was collected at 500 to 545 nm. A maximum intensity projection was generated using ImageJ software (version 1.49; http://imagej.nih.gov/).

### Transmission electron microscopy

Preparation of the samples for transmission electron microscopy (TEM) followed the procedure used by Płachno et al. (2014). The sections were examined using a Hitachi H500 electron microscope at 75 kV in the Faculty of Biology and Environmental Protection, University of Silesia in Katowice.

## Results

### Ovules containing a mature embryo sac

**Fig. 2:**
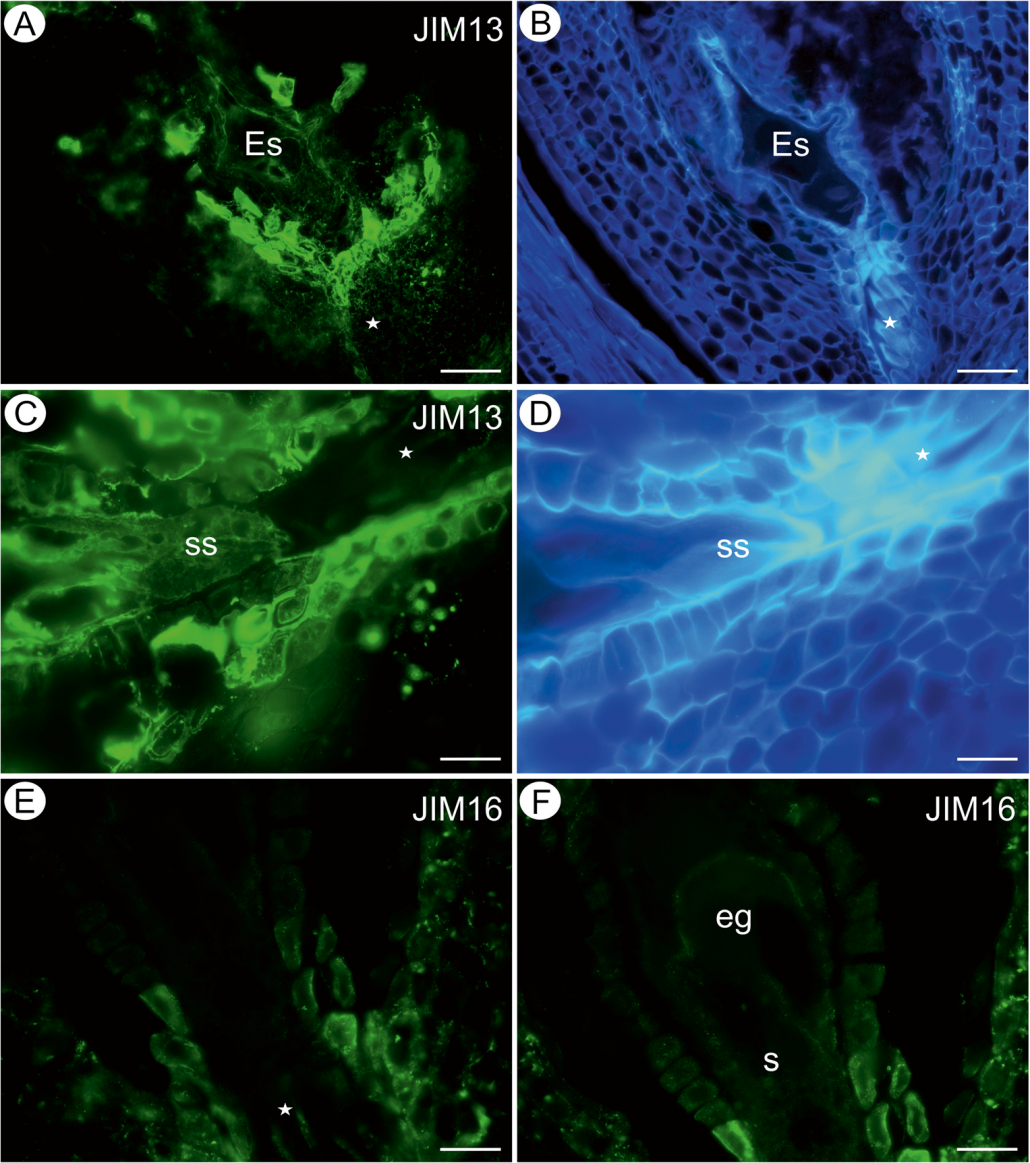
Arabinogalactan protein detection in an ovule containing a mature embryo sac (*Es*). **a** Presence of the JIM13 antibody in micropylar transmitting tissue cells (*star*), *bar* = 50 μm. **b** The same section as **a**, cellulose visualisation, *bar* = 50 μm. **c** Presence of the JIM13 antibody in synergids (*ss*; *white star* points to the transmitting tissue), *bar* = 20 μm. **d** The same section as **c**, cellulose visualisation (*ss* synergids; *white star* points to the transmitting tissue), *bar* = 20 μm. **e**, **f** Presence of the JIM16 antibody in micropylar transmitting tissue cells (*star*; *eg* egg cell, *s* synergid), *bar* = 20 μm

The AGP detected by the JIM13 antibody was present in the cytoplasm (the term “cytoplasm” in the whole manuscript must be understood as an abbreviation form of “localization within the cytoplasm endomembrane system”, not directly in the cytoplasm; but as an analysis was performed on the light microscope level, determination of which kind of membranes was involved was not possible; moreover, this statement indicates the cytoplasmic localisation in the close proximity to the plasma membrane because the vicinity of the cell wall suggests such interpretation) and cell wall of the integument cells near the egg apparatus, the integument cells surrounding the micropylar canal (Fig. 2a, b), in the central cell and in the synergids (Fig. 2c, d; sup. mat. Fig. 1A, B). The JIM13 was detected in the transmitting tissue cells but in a much lower amount (Fig. 2a; sup. mat. Fig. 1). In contrast, the AGP recognised by the JIM16 antibody was present only in the parietal layer of cytoplasm of the integument cells surrounding the micropylar canal, the apex of the synergids and the transmitting tissues, where this epitope was also detected in cell walls (Fig. 2e, f). Moreover, the JIM16 antibody was present in the borders between the cells of the egg apparatus and the central cell (Fig. 2f). The AGP recognised by the JIM8 antibody was not detected in any of the analysed cells in both developmental stages.

**Fig. 3:**
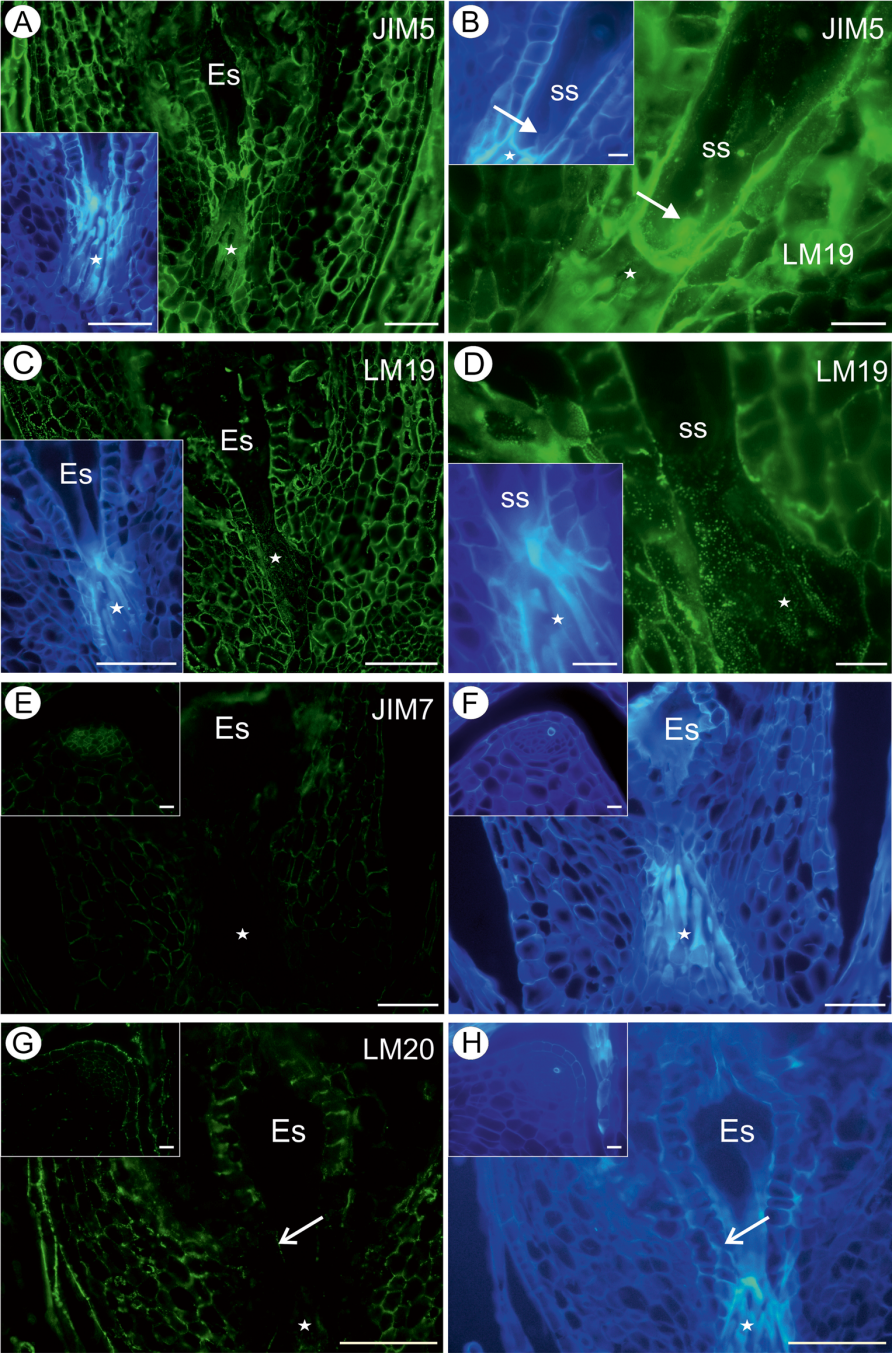
Pectin detection in an ovule containing a mature embryo sac (*Es*). **a** Presence of the JIM5 antibody in micropylar transmitting tissue cells (*star*), cellulose visualisation (*inset*), *bar* = 50 μm. **b** Presence of the JIM5 antibody in synergids (*ss* synergids; *white arrow* indicates the synergid filiform apparatus) and micropylar transmitting tissue cells (*star*), cellulose visualisation (*inset*), b*ar* = 20 μm. **c** Distribution of the LM19 antibody in embryo sac and synergids (*ss*; *inset*) and in micropylar transmitting tissue cells (*white star*), *bar* = 50 μm. **d** The same section as **c**, cellulose visualisation, *bar* = 50 μm. **e** Distribution of the JIM7 antibody. Note the lack of signal in the transmitting tissue (*white star*), but the presence of signal in the integumentary cell walls and the chalazal pole (*inset*), *bar* = 50 μm. **f** The same section as **e**, cellulose visualisation, *bar* = 50 μm. **g** Distribution of the LM20 antibody (*open arrow* points to the outer periclinal wall of the tapetum cells). Note the weak signal in the transmitting tissue (*white star*), but the presence of signal in the integumentary cell walls and the chalazal pole including the epidermis (*inset*), *bar* = 50 μm. **h** The same section as **g**, cellulose visualisation, *bar* = 50 μm; *bar on all the insertions* = 10 μm

The pectic epitope recognised by the JIM5 antibody was abundantly present in the walls of different integument cells, the extracellular matrix of the transmitting tissue cells, although it was absent from the cytoplasm (Fig. 3a). In some cells, this epitope was also present in the cytoplasm of the integument cells surrounding the micropylar canal, the apex of the synergids and the filiform apparatus of synergids (Fig. 3b). Always such a localisation was in the close proximity to the cell wall (parietal layer of cytoplasm). The LM19 epitope was present mostly in the cell walls of the integument tissues, and the fluorescence was especially strong in the integument tissues near the egg apparatus and in transmitting tissues (Fig. 3c, d). A low amount of this epitope was noted also in the cytoplasmic part of the cell (Fig. 3c, d).

**Fig. 4:**
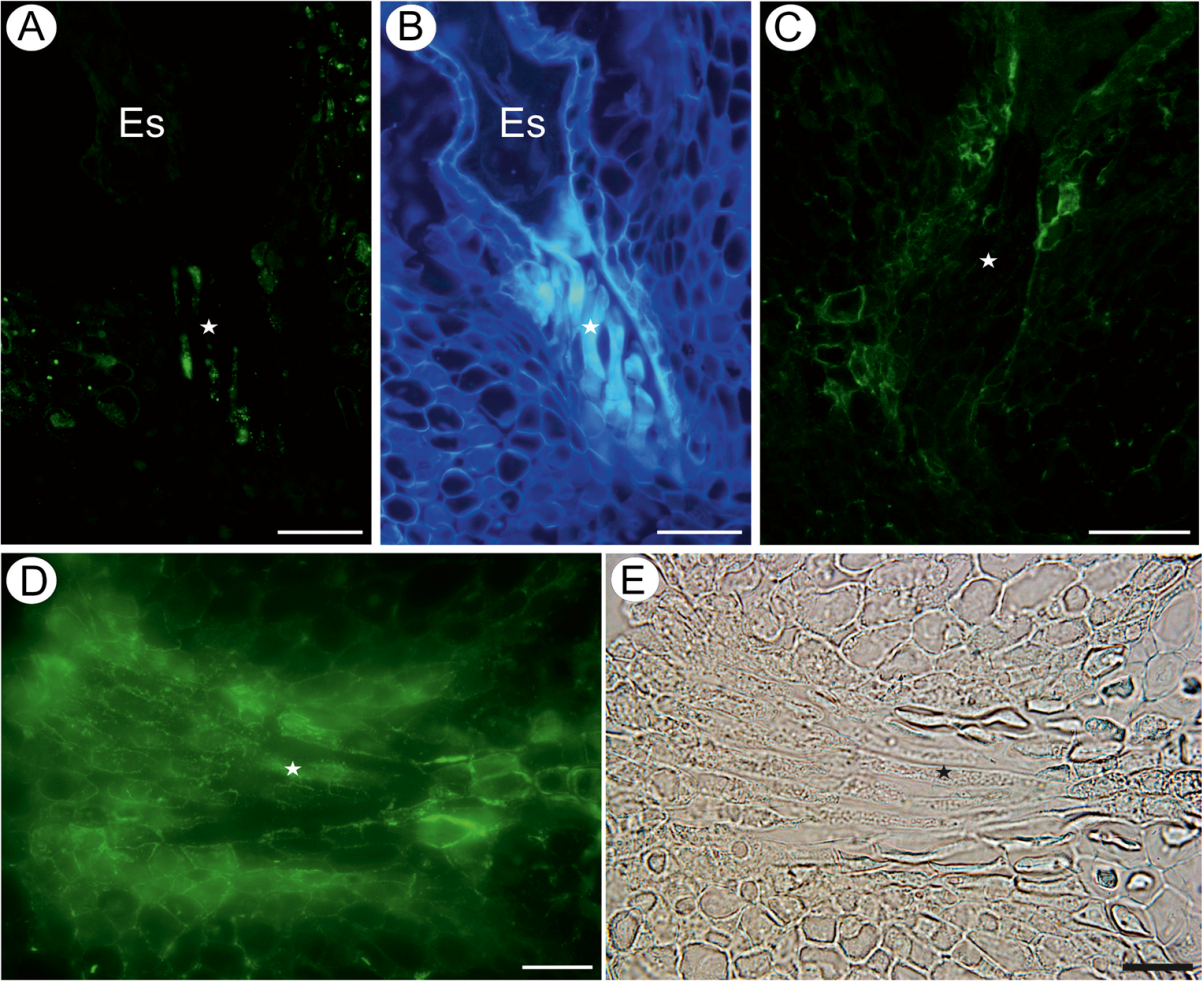
Hemicellulose detection in an ovule containing a mature embryo sac (*Es*). **a** Section labelled with the LM21 antibody, micropylar transmitting tissue cells, *bar* = 50 μm. **b** The same section as **a**, cellulose visualisation, *bar* = 50 μm. **c**, **d** Sections labelled with the LM25 antibody, *bar* = 50 and 20 μm, respectively. **e** The same as **d** but seen in a differential interference contrast, micropylar transmitting tissue cells (*star*), *bar* = 20 μm

Pectic epitope recognised by the JIM7 antibody was detected in most of the ovule tissues except from the transmitting tissue and the embryo sac (Fig. 3e, f). The LM20 epitope was present in the cell walls of the integumentary tapetum near the central cell of the embryo sac and in the transmitting tissue cells (Fig. 3g, h); however, it was absent from the integumentary cells surrounding the egg apparatus (Fig. 3g, h). The LM8 antibody was not detected in any of the analysed cells independent on the developmental stage. The hemicellulose epitope recognised by the LM21 antibody was detected mostly in the walls of transmitting tissue cells and cytoplasm of some integument cells near the embryo sac and micropylar canal (Fig. 4a, b). The LM25 epitope was present in the integumentary epidermis near the apex of the embryo sac, and the fluorescence was very intense (Fig. 4c). Fluorescence signal was also detected in the cytoplasm and the walls of the transmitting tissue cells (Fig. 4d, e).

### A young seed containing an embryo and endosperm

**Fig. 5:**
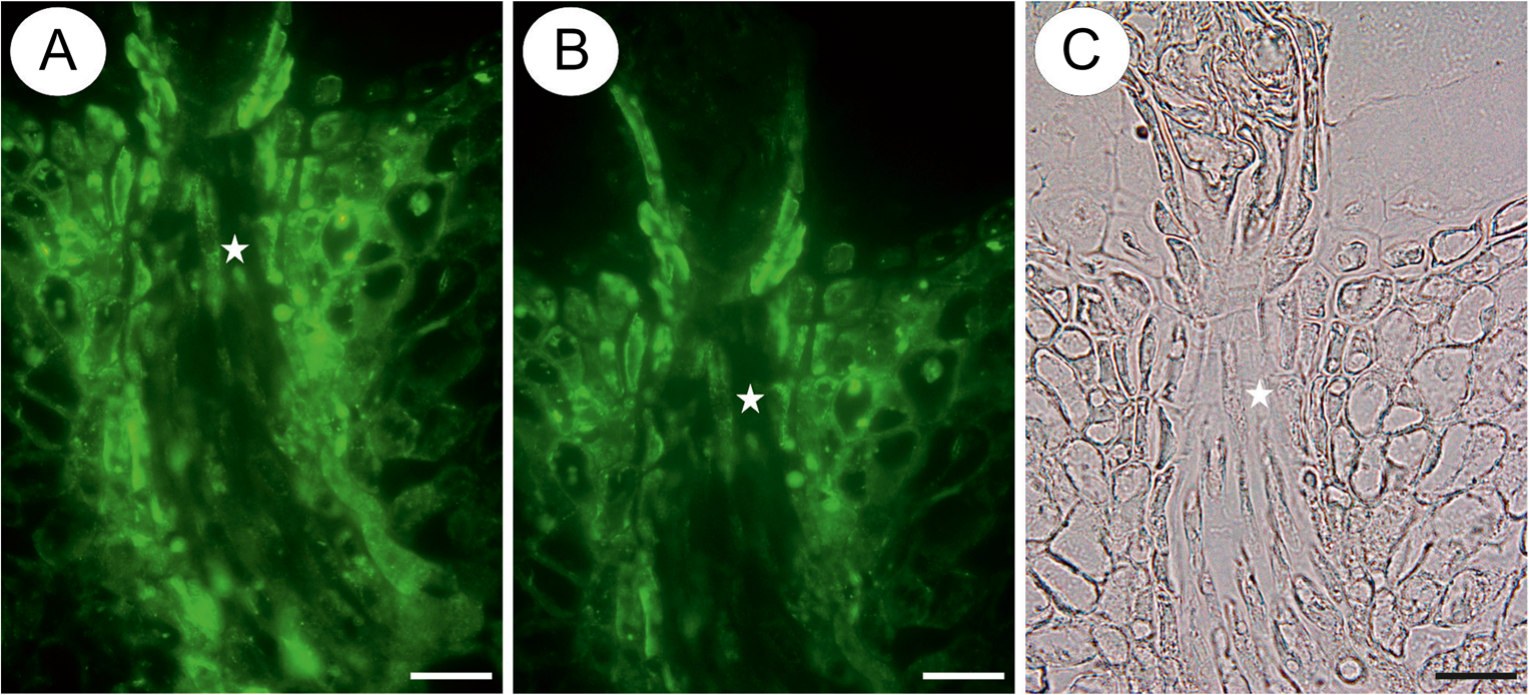
Distribution of arabinogalactan proteins in a seed containing an embryo and endosperm. **a**, **b** Distribution of the JIM16 antibody in micropylar transmitting tissue cells, *bar* = 20 μm. **c** The same as **b** but seen in a differential interference contrast (*star* micropylar transmitting tissue cells), *bar* = 20 μm

The AGP epitope recognised by the JIM16 antibody was present in the transmitting tissue cell cytoplasm and in the integumentary cells bordering the micropylar canal (Fig. 5a–c). An intense fluorescence signal was detected in the integumentary epidermal cells of the border of the endosperm (at the micropylar pole; Fig. 5b). The distribution of the JIM13 epitope was the same as in the former described developmental stage (data not shown).

**Fig. 6:**
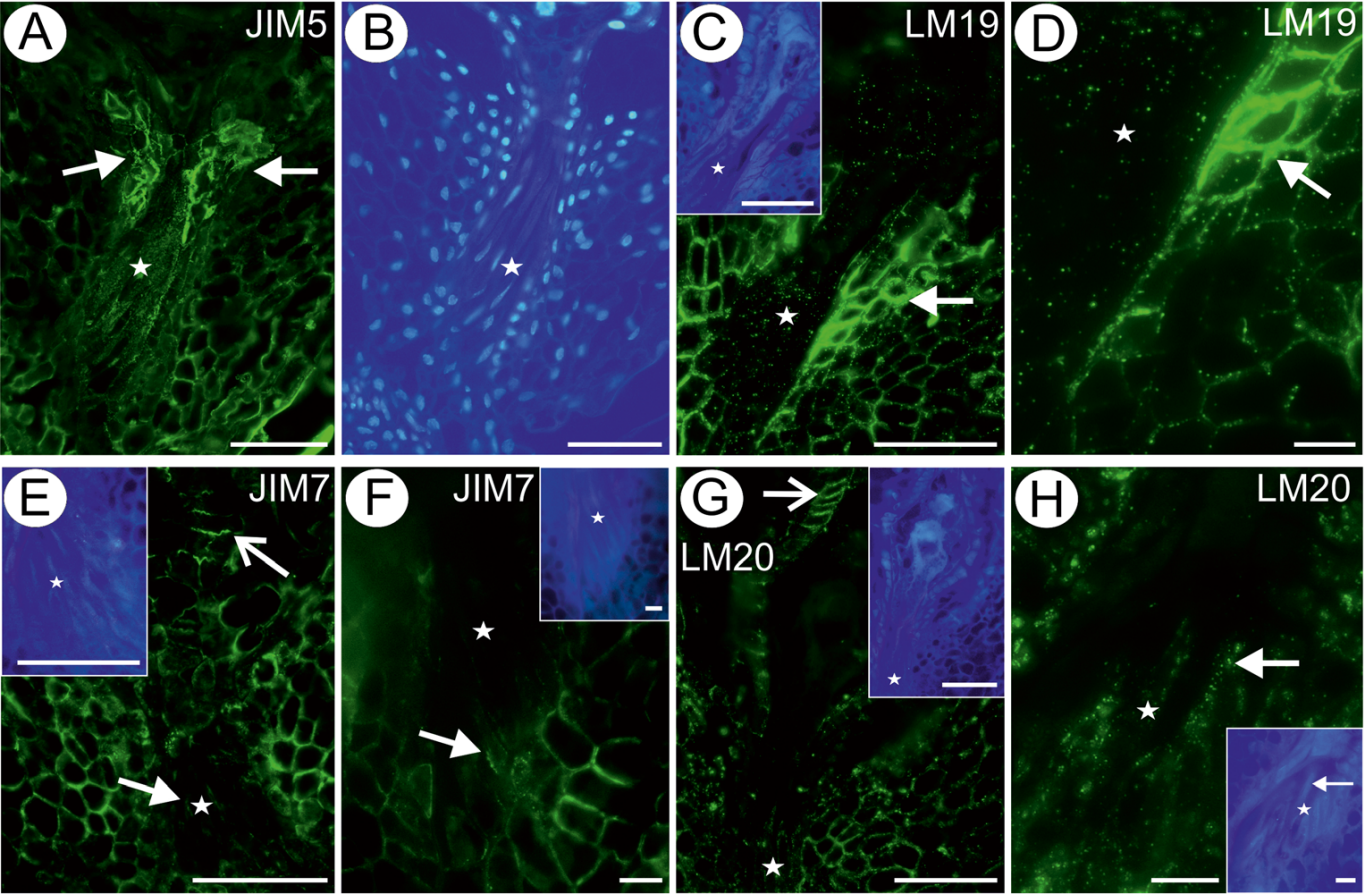
Pectin detection in a young seed containing an embryo and endosperm. **a** Distribution of the JIM5 antibody in micropylar transmitting tissue cells and in the integument cells (*arrows*) near the synergids, *bar* = 50 μm. **b** The same as **a** but nuclei visualisation, DAPI staining, *bar* = 50 μm. **c** Distribution of the LM19 antibody in micropylar transmitting tissue cells; *arrow* points to the cell walls of some integumentary cells in the vicinity of micropylar transmitting tissue cells, *bar* = 50 μm; *inset* cellulose visualisation, *bar* = 50 μm. **d** Higher magnification of **c**, *bar* = 10 μm. **e** Distribution of the JIM7 antibody. Note the weak signal in the transmitting tissue (*white star*), but the stronger signal in the integumentary cell walls (*open arrow*); *arrow* points to the signal located in the cell wall of transmitting tissue (*inset* cellulose visualisation; *bar* = 50 μm), *bar* = 50 μm. **f** The presence of the JIM7 antibody in the walls (*arrow*) of transmitting tissue cells (*star*); *inset* cellulose visualisation, *bar* = 10 μm. **g** Distribution of the LM20 antibody (*open arrow* points to the anticlinal walls of the inner integumentary epidermal cells). Note the presence of this pectic epitope in the walls of different ovule tissues, but weaker signal in the transmitting tissue cells (*star*). *Inset* cellulose visualisation, *bar* = 50 μm. **h** Another example of the presence of the LM20 antibody in transmitting tissue cell walls (*arrow*). *Inset* cellulose visualisation, *bar* = 10 μm

The pectic epitope recognised by the JIM5 antibody was abundantly present in the walls of different integument cells, especially in those surrounding the micropylar canal, and in the transmitting tissue cells (Fig. 6a, b). The LM19 epitope occurred in most of the cell walls of the integument tissue cells especially near the micropylar canal (Fig. 6c, d). This epitope was detected in the walls and extracellular matrix of transmitting tissue cells (Fig. 6c, d). The pectic epitope recognised by the JIM7 antibody was present in walls of integument cells and in a very low amount in transmitting tissues cell walls (Fig. 6e, f). The LM20 epitope was detected in the walls of the transmitting tissue cells and in the parenchyma of the integument (Fig. 6g, h).

### TEM

**Fig. 7:**
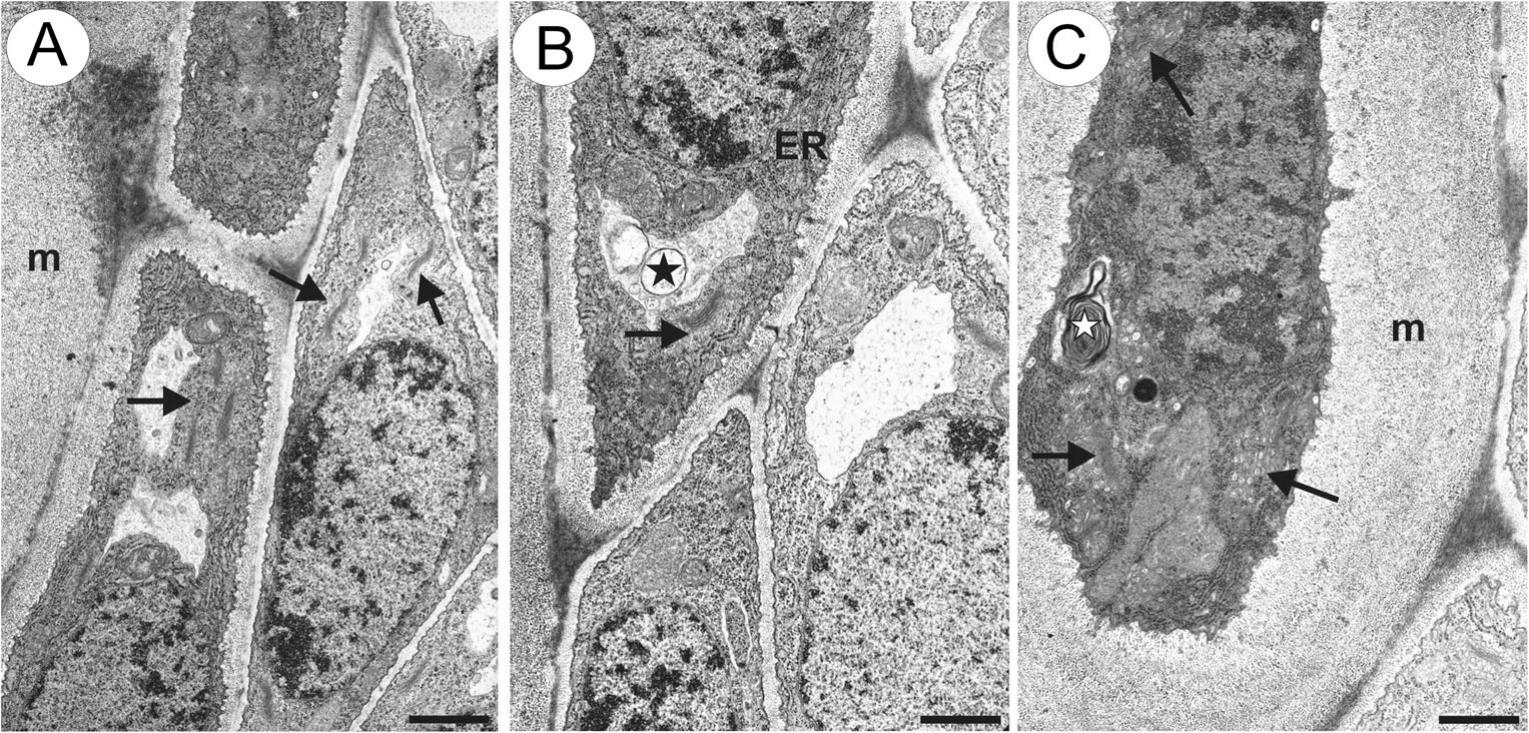
Ultrastructure of integument cells in the micropylar pole near the egg apparatus. **a**, **b** Cells that border mucilage cells, note the cytoplasm rich in ribosomes, well-developed rough endoplasmic reticulum (*ER*) and non-hypertrophied dictyosomes (*arrows*). Vacuole with small vesicles (*star*), mucilage layer (*m*), *bar* = 1.3 and 1 μm. **c** Mucilage cell, note the mucilage layer (*m*) between the plasmalemma and cell wall, hypertrophied dictyosomes (*arrows*), membranous vacuolar inclusion (*star*), *bar* = 0.85 μm

Because we observed the strong labelling of the integument cells at the micropylar pole near the egg apparatus with the JIM13 antibody, the ultrastructure of these cells was studied (Fig. 7a–c). Some of these cells did not produce mucilage and were characterised by dense cytoplasm filled with numerous ribosomes, non-hypertrophied dictyosomes and small vacuoles that sometimes contained some vesicles (Fig. 7a, b). Other cells that produced mucilage had a very dense cytoplasm with hypertrophied dictyosomes (Fig. 7c).

## Discussion

The results presented here concern the distribution of AGP, pectic and hemicellulose epitopes in the reproductive structures of *Taraxacum* and indicate some spatio-temporal differences and similarities in ovule and seed.

### AGPs

Most of our present knowledge concerning the involvement of AGPs in developmental processes comes from studies on somatic embryogenesis and root organogenesis (van Hengel et al. 2002; Rumyantseva 2005; Wilson et al. 2015; Šamaj et al. 1999; Tchorbadjieva 2005; Seifert and Roberts 2007). Results shown in the current studies broaden the knowledge on the putative involvement of AGPs in ovule and seed development on the example of *Taraxacum*. Arabinogalactan proteins were detected in the ovules of species from different evolutionary lines of angiosperms, e.g. in the early divergent angiosperms Nymphaeales (*Trithuria*, Hydatellaceae; Costa et al. 2013) and Austrobaileyales (*Illicium*, *Trimenia*, Sage et al. 2009) as well as in core eudicots (e.g. *Brassica*, Pennell et al. 1991; *Sinapis*, Chudzik et al. 2005a, b; *Arabidopsis*, Coimbra et al. 2007; *Olea*, Suárez et al. 2013) and monocots (*Galtonia* and *Galanthus*, Chudzik et al. 2005b).

AGPs were detected at the micropylar ovule pole in the tissues lying on the pathway of pollen tube growth. In the crassinucellate ovule type, AGPs were recorded in the micropylar nucellar tissue and the integuments lining the pollen tube growth pathway (Coimbra and Salema 1997; Chudzik et al. 2005a, b). In the tenuinucellate ovule type, AGPs were observed in the integument cells that define the micropyle as in *Arabidopsis* (Coimbra et al. 2007) or in the micropylar canal as in *Galanthus* (Chudzik et al. 2005b). In the tenuinucellate ovule of *Taraxacum*, the intense labelling of JIM13 was seen in the integument cells surrounding the micropylar canal in contrast to the micropylar transmitting tissue cells, which were only slightly labelled. However, AGP epitope recognised by the JIM16 antibody was present in the parietal layer of cytoplasm of the micropylar transmitting tissue cells. The ultrastructure of the integument cells surrounding the micropylar canal was previously analysed by Płachno and co-authors (2015a). The exocytosis of various vesicles was observed in these cells. Thus, given the occurrence of AGP (both JIM13 and JIM16 epitopes) and the ultrastructure data, it is suggested that these cells may also participate in interactions with the pollen tubes (in sexual *Taraxacum* and, in rare cases, in apomicts, see Małecka 1973; van Baarlen et al. 2002; Mártonfiová 2006). An interesting result presented here is the strong labelling of integument cells near the egg apparatus with the JIM13 antibody. These cells border the fully developed integument mucilage cells (Płachno et al. 2015a), and their cytoplasm was rich in ribosomes.

In all of the amphimictic species studied, a strong accumulation of the arabinogalactan proteins (both the JIM13 and JIM8 epitopes or only JIM13) was recorded in the filiform apparatus of synergids (Pennell et al. 1991; Chudzik et al. 2005a, b; Coimbra and Salema 1997; Coimbra et al. 2007). In contrast, we did not observe such an accumulation of arabinogalactan proteins (JIM13) in the filiform apparatus of *Taraxacum* synergids. However, weak labelling was observed in the synergid walls (including the filiform apparatus) and the cytoplasmic compartments. There can be a few explanations for this phenomenon, e.g. the structure of the filiform apparatus in *Taraxacum* or the apomictic mode of reproduction. Moreover, an accumulation of this AGP epitope may be correlated with pollination and the growth of pollen tubes in the style; e.g. AGP epitopes were not detected in open but unpollinated flowers of *Galanthus* ovules (Chudzik et al. 2005a, b). In *Taraxacum* (Płachno et al. 2014) as in other Asteraceae (e.g. *Helianthus*, Newcomb 1973; Yan et al. 1991), the thickened adjacent walls of both synergids build the filiform apparatus. This Asteraceae type of filiform apparatus is different from the classical filiform apparatus, which comprises an elaborate system of highly branched projections (Huang and Russell 1992) and in which a strong accumulation of arabinogalactan protein was found. Unfortunately, to date, only two species from the Asteraceae family have been studied to analyse the occurrence of AGPs in the embryo sac and ovule. According to Chudzik et al. (2010), in the mature ovules in amphimictic *B. perennis*, the AGPs that were recognised by the JIM13 and JIM16 antibodies were distributed in the cell walls of the embryo sac and egg apparatus, but the MAC207 epitope did not occurred in the female gametophyte. In the apomict *C. juncea*, the AGP epitope recognised by the JIM13 antibody was not recorded in the micropylar canal or the mature embryo sac (Chudzik et al. 2005a). Kościńska-Pająk and co-authors (2005), Kościńska-Pająk (2006), Kościńska-Pająk and Bednara (2006) and Chudzik et al. (2005a) suggested that the synergids in obligatory apomicts are in the juvenile phase and do not show the secretory activity that is characteristic of amphimictic plants. In contrast to *Chondrilla*, the cytoplasm in the apomictic *Taraxacum* synergids contained arabinogalactan proteins labelled by JIM13 and JIM16 antibodies. Pennell et al. (1991) showed that arabinogalactan proteins occurred in Golgi-derived vesicles in the synergids of *Brassica*. Thus, we propose that AGP recognised by the JIM16 antibody may be a useful marker for the borders between the cells of the egg apparatus and the central cell. The whole embryo sac was labelled with JIM13 in *Sinapis alba* (Chudzik et al. 2005b), while not only the synergids but also the cell walls of the central cell were labelled with JIM13 in *Arabidopsis* (Coimbra et al. 2007). We made a similar observation in the case of *Taraxacum*, in which the arabinogalactan proteins occurred in the central cell cytoplasm (localisation within the cytoplasm endomembrane system) and walls.

To summarise, the AGPs that occur along the pathway of pollen tube growth in amphimictic (sexual) plants may also occur in similar places in apomicts as we showed in *Taraxacum*. The relationship between the occurrence of AGPs and the pathway of pollen tube growth is very conservative in evolutionary terms because AGPs have also been detected between the nucellus cells in gymnosperm ovules (Rafińska and Bednarska 2011).

### Pectins

Pectins are diverse wall chemical components (Caffall and Mohnen 2009; Palmer et al. 2015) and are considered to be determinants in plant development and plant tissue differentiation (e.g. Wolf et al. 2009). It has been well documented that homogalacturonan (HG) plays an important role in pollen-stigma and pollen tube-pistil interactions in flowering plants (see Sage et al. 2009; Niedojadło et al. 2015 and discussion therein). In amphimictic plants, there are pollination- and fertilisation-induced changes in the occurrence of highly (JIM7) and low (JIM5) methyl-esterified pectins in the pistil transmitting tract, the micropyle of the ovule and also in the apoplast of female gametophyte cells (Lenartowska et al. 2001; Śnieżko and Chudzik 2003; Suárez et al. 2013; Niedojadło et al. 2015). In contrast to amphimictic plants (e.g. *Galanthus*, Śnieżko and Chudzik 2003; *Olea*, Suárez et al. 2013; *Hyacinthus*, Niedojadło et al. 2015), we did not observed abundant occurrence of highly methyl-esterified HG labelled by the JIM7 antibody in either the micropyle or the synergids of apomictic *Taraxacum*. Neither of the two HG domains (recognised by JIM7 and JIM5 antibodies) were recorded in the micropylar part of the embryo sac nor in the cytoplasm of the synergids in apomict *C. juncea* (Kościńska-Pająk et al. 2005; Kościńska-Pająk 2006). In transmitting tissues of *Taraxacum*, the JIM5 epitope was present in both analysed developmental stages, but the JIM7 epitope was detected only in the stage of the young seed. These pectins also occurred in *Taraxacum* in the filiform apparatus of the synergids. There is rearrangement of the HG presence in transmitting tissue during ovule maturation and the stages of pollination in amphimictic plants. In apomictic *Taraxacum*, the similar pattern of low methyl-esterified HG characterised both developmental stages. The presence of high methyl-esterified pectins appeared with the more advanced stage of development.

Differences which we found in the presence of pectic epitopes recognised by JIM5, JIM7, LM19 and LM20 antibodies depended most probably on the range of epitopes they recognised. In general, some similarities between these epitope pairs are described (Verhertbruggen et al. 2009). In our case, it is visible that JIM7 and LM20 epitopes occur in the similar manner in the parenchymatic tissue of the ovule or in the integument tapetum (Figs. 3e, g and 6e, g); however, differences are found in the epidermal tissue of ovule where LM20 occurs abundantly in contrast to JIM7 (Fig. 3e and inset) or in transmitting tissue (Figs. 3 and 6). JIM5 and LM19 occurrence is also consistent with two differences in epitope distribution: the LM19 epitope occurs often in a more punctate manner than JIM5, and JIM5 occurs more abundantly in the walls of transmitting tissue (compare Figs. 3 and 6).

The occurrence of pectin epitopes with a low degree of esterification in the cytoplasm is rather unexpected as HG is synthesised in a highly methyl-esterified form in the Golgi apparatus (Bárány et al. 2010). Such result may indicate the artefacts or the presence of pectin detected with the use of JIM5 antibody within the cytoplasmic compartments. The presence of JIM5 epitope in the cytoplasm is seldom described in the literature. In the case of *Arabidopsis thaliana* protoplasts, the presence of JIM5 antibody within the cytoplasm was described together with the wall-associated kinases (Kohorn et al. 2006). Another example comes from studies on stylar transmitting tissues in *Brugmansia suaveolens* (Hudák et al. 1993), where it was shown on the transmission electron microscopy level that pectic epitope recognised by the JIM5 antibody was present in the cytoplasmic membrane system, mostly in ER, AG and multivesicular bodies, and the distribution changed depending on the developmental stage. Thus, the results presented here may be another example of the presence of low methyl-esterified pectins not only in the cell wall but also in the cytoplasm compartments. Another explanation of this result is that both JIM5 and JIM7 antibodies have a rather big set of common elements (Knox et al. 1990; Willats et al. 2000; Clausen et al. 2003). Therefore, the JIM5 antibody may also detect some pectic epitopes recognised by the JIM7 antibody.

### Hemicelluloses

Hemicelluloses are polysaccharides that are involved in the cross-linking of cellulose microfibrils (Scheller and Ulvskov 2010; Palmer et al. 2015). The hemicellulose content and deposition within the cell wall change, and it is postulated that this chemical component plays a specific role in the wall matrices during cell wall formation and development (Palmer et al. 2015). Studies with the more recently generated LM25 xyloglucan antibody and the LM21 heteromannan antibody showed a differential presence of these hemicelluloses in the cell walls of wheat and rice undergoing cellularisation in the syncytial endosperm (Palmer et al. 2015). The analyses presented here showed a differential distribution of xyloglucan and heteromannan since the first one was detected in the integumentary epidermis near the apex of the embryo sac and in the cytoplasm and extracellular matrix of the transmitting tissue cells, and the second one was detected in the walls of the transmitting tissue cells and in some integument cells (where it may also occur in the cytoplasm) near the embryo sac and the micropylar canal. To date, the distribution of hemicelluloses has only been analysed on the example of barley, wheat and rice during cellularisation (Wilson et al. 2012; Palmer et al. 2015) and it is difficult to compare our results with the literature. It is postulated (Palmer et al. 2015) that hemicellulose polysaccharides may influence the plasticity of the cell wall, thus allowing the cells to respond to the rapid changes during the development of the cell wall or that they may represent a more easily digestible storage medium than microfibril cellulose (Palmer et al. 2015), which in the case of *Taraxacum* requires further studies.

In conclusion, our findings reveal that an accumulation of arabinogalactan proteins (AGPs) and some pectic epitopes occurred in the micropylar transmitting tissue of apomictic *Taraxacum*, which is similar to the micropylar ovule tissues of other sexual plant species. There is rearrangement of HG during ovule maturation and the stages of pollination in amphimictic plants. This is the first time that data about the occurrence of hemicelluloses (LM25 and LM21) in the reproductive structure of flowering plants are presented in the literature. Moreover, the accumulation of both, low methyl-esterified HG (JIM5, LM19) and AGP (JIM13), occurred in the integument cells surrounding the micropylar canal near the synergids. Such a diverse description of the presence of different pectic, AGP and hemicellulose epitopes is presented in a spatio-temporal manner on the example of apomict plants for the first time (at least to authors’ knowledge).

## Electronic supplementary material

Below is the link to the electronic supplementary material.

ESM 1 Supplementary material – Fig 1. Reconstruction from a confocal microscope. JIM13 antibody distribution in an ovule containing a mature embryo sac. A-B. Micropylar part of an ovule. Note strong labelling with JIM13 of integument cells at the micropylar pole near egg apparatus (red star) and cells of micropylar canal (arrows). egg cell-eg, synergids-ss, micropylar transmitting tissue cells-white star, A- bar = 50 μm, and B- bar = 10 μm. (JPG 4864 kb)
