# Supplementary figures and images for: Immunodetection of some pectic, arabinogalactan proteins and hemicellulose epitopes in the micropylar transmitting tissue of apomictic dandelions (Taraxacum, Asteraceae, Lactuceae)
Source: Protoplasma. 2016 May 6;254(2):657–68. doi: 10.1007/s00709-016-0980-0 (PMC5309284; doi:10.1007/s00709-016-0980-0)

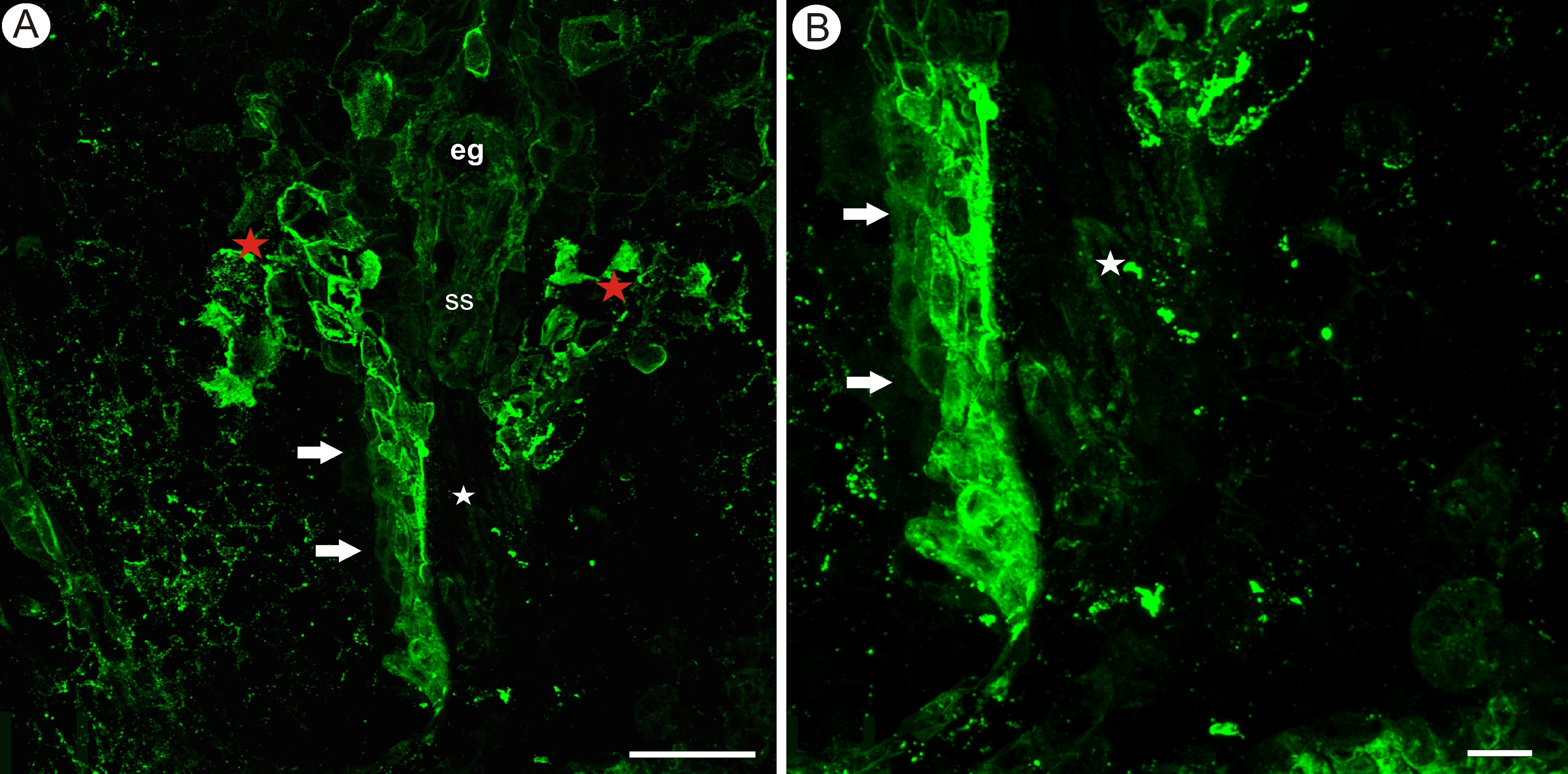

Supplement: Supplementary file 1 — Supplementary material – Fig 1. Reconstruction from a confocal microscope. JIM13 antibody distribution in an ovule containing a mature embryo sac. A-B. Micropylar part of an ovule. Note strong labelling with JIM13 of integument cells at the micropylar pole near egg apparatus (red star) and cells of micropylar canal (arrows). egg cell-eg, synergids-ss, micropylar transmitting tissue cells-white star, A- bar = 50 μm, and B- bar = 10 μm. (JPG 4864 kb) [file 709_2016_980_MOESM1_ESM.jpg]
